# The LPA-CDK5-tau pathway mediates neuronal injury in an in vitro model of ischemia-reperfusion insult

**DOI:** 10.1186/s12883-022-02694-2

**Published:** 2022-05-02

**Authors:** Yaya Wang, Jie Zhang, Liqin Huang, Yanhong Mo, Changyu Wang, Yiyi Li, Yangyang Zhang, Zhaohui Zhang

**Affiliations:** Department of Neurology, Renmin hospital of Wuhan University, Wuhan, 430060 China

**Keywords:** LPA, CDK5, Tau, Ischemia reperfusion

## Abstract

Lysophosphatidic acid (LPA) is a common glycerol phospholipid and an important extracellular signaling molecule. LPA binds to its receptors and mediates a variety of biological effects, including the pathophysiological process underlying ischemic brain damage and traumatic brain injury. However, the molecular mechanisms mediating the pathological role of LPA are not clear. Here, we found that LPA activates cyclin-dependent kinase 5 (CDK5). CDK5 phosphorylates tau, which leads to neuronal cell death. Inhibition of LPA production or blocking its receptors reduced the abnormal activation of CDK5 and phosphorylation of tau, thus reversing the death of neurons. Our data indicate that the LPA-CDK5-Tau pathway plays an important role in the pathophysiological process after ischemic stroke. Inhibiting the LPA pathway may be a potential therapeutic target for treating ischemic brain injury.

**Supplementary Information:**

The online version contains supplementary material available at 10.1186/s12883-022-02694-2.

## Introduction

LPA is a small molecule with a simple glycerolipid structure. As an important extracellular molecule, LPA regulates a series of physiological and pathological processes by binding to its receptors. Different receptors are distributed in different tissues and mediate specific biological effects [1]. Among LPA receptors, LPA receptor 1(LPA1) and LPA receptor 2(LPA2) are mainly expressed in the nervous system and participate in diseases of the central nervous system, such as ischemic stroke, brain trauma, congenital hydrocephalus, multiple sclerosis, and developmental diseases of the nervous system [2–4]. Phospholipase A1 (PLA1), phospholipase A2 (PLA2) and autotaxin (ATX) are three main enzymes that mediate the production of LPA [5]. ATX is the most well studied enzyme that mediates LPA production [6]. It has been reported that ATX-mediated LPA production is involved in neuropathic pain caused by nerve injury [7] and dorsal root demyelination [8]. This evidence suggests that LPA receptor inhibitors and ATX inhibitors may be useful for treating LPA-induced nerve injury. Under some pathological conditions such as atherosclerosis, brain injury, spinal cord injury, neuralgia, and neuropsychiatric disorders, the concentration of LPA in the blood increases [9]. The concentrations of LPA and its receptors in the blood of patients with ischemic stroke were increased [10, 11]. Our previous studies also found elevated serum LPA levels in patients with ischemic stroke and in models of middle cerebral artery occlusion (MCAO) in rats [12]. These studies indicate that LPA plays an important role in nerve injury in ischemic stroke. However, the molecular mechanisms by which LPA regulates pathological changes after ischemic stroke remains unclear.

CDK5 is a member of the Ser/Thr cyclin-dependent kinases (CDKs) family. CDK5 is a catalytic subunit with neuron-specific activity that functions by binding to the neuron-specific catalytic factors p35 or p25 [13, 14]. P35 can be cut into p25 by calpain. P25 further activates CDK5 [15, 16]. It has been reported that abnormal CDK5 activation is involved in the pathogenesis of stroke [17, 18]. Abnormal CDK5 activation has been found in the MACO mouse model [19]. CDK5 phosphorylates tau and participate the onset of Alzheimer’s patients [20]. There are 3-4 major CDK5 phosphorylation sites on the tau protein, especially Ser202 and Thr205 [21, 22]. It has also been reported that cerebral ischemia induces tau hyperphosphorylation [23]. Thus, we hypothesized that CDK5, which is abnormally activated after stroke, phosphorylates tau and mediates neuronal cell death. Since CDK5 is abnormally activated and LPA is elevated after cerebral ischemia, we hypothesized that LPA may be related to CDK5, which may lead to neuronal damage during ischemic stroke.

## Materials and methods

### Materials

CDK5 antibody (10430-1-AP), GAPDH antibody (21000453), Caspase-3 antibody (66470-2-Ig), Map2 antibody (17490-1-AP) and CDK5 inhibitor (roscovitine) were purchased from Proteintech. 18:1 LPA (L7260) was obtained from Sigma-Aldrich. One-step TUNEL apoptosis assay kit(C1080) and μ-calpain antibody (AC012-1) were purchased from Beyotime. Cell count kit 8 (CCK-8, HY-K0301), LPA1 receptor antagonist (AM095, HY-16039) and LPA2 receptor antagonist (HY-18075) were purchased from MedChemExpress. Autotaxin inhibitor (PF8380) was obtained from APExBIO. AT8 antibody (MN1020) was obtained from Thermo. The ELISA kit (MU30816) was obtained from Bio-swamp.

Common cell culture medium was composed of DMEM 500 mL, 5 mL cyan- streptomycin and 50 mL bovine serum. The neuronal medium consisted of Nrobase 500 mL, B27 10 mL, glutamine 5 mL and cyan-streptomycin 5 mL. The LPA reserve solution was prepared by dissolving the LPA in phosphate-free buffer saline (PBS) with a pH of 7.2, which was free of calcium and magnesium. 5 mg LPA was dissolved in about 11.45 ml PBS to obtain 1 mM LPA reserve solution. The glucose ECS: the solvent is sterile water, the solute include glucose, NaCl, KCl and NaHCO_3_. The glucose-free ECS has no solute of glucose as compared to sugar-free ECS.

### Cell culture

SY5Y cells were cultured at 37 °C in a 5% CO2/95% air atmosphere from CCFTCC. Mouse cortical neurons from Wt embryos, the embryos were about 18 days old, according to the previous description [24, 25], were cultured at 37 °C in a 5% CO_2_ incubator for 10 days. Treatment: Cortical neurons were treated with LPA (0, 4, 8, and 12 μM) for 4 h; DMSO (0.5 μM), LPA1 receptor inhibitor (0.5 μM), LPA2 receptor inhibitor (0.05 μM) and CDK5 inhibitor (0.5 μM) were added for a 2 h pretreatment, and then LPA (8 uM/L) was added for a 4 h treatment. SY5Y cells were treated with LPA (0, 30, 60, and 90 μM) for 24 h. DMSO (5 μM), LPA1 receptor inhibitor (5 μM), LPA2 receptor inhibitor (5 μM) and CDK5 inhibitor (5 μM) were added for a 2 h pretreatment, followed by LPA (60 μM) treatment for 24 h. In oxygen and glucose deprivation (OGD) experiments, ATX inhibitor (0.5 μM) were added for a 2 h pretreatment in cortical neurons, ATX inhibitor (5 μM) were added for a 2 h pretreatment in SY5Y cells, and then the cells were incubated in a glucose-free medium at 37 °C in a hypoxic environment for 4 h (cortical neurons) or 24 h (SY5Y cells). The cells were then reperfused for 24 h.

### Cell viability test

The CCK-8 kit was used to determine the viability of cortical neurons and SY5Y cells. Cortical neurons and SY5Y cells were seeded on 96-well plates (10,000 cells/well). CCK-8 reagent (10 μl/well) was added to the wells, and incubated at 37 °C with 5%CO_2_ for 1 h. The cells were placed in an plate reader and read at 450 nm. The Cell Counting Kit-8 (CCK-8) (Dojindo) was used to evaluate cell viability according to the instructions, and the cell viability = [(As-Ac)/ (Ac-Ab)] × 100%. As was the experimental group, Ac was the control group, and Ab was the blank group.

### TUNEL staining

Apoptotic DNA fragmentation was examined using the One-step TUNEL apoptosis assay kit according to the manufacture’s protocol. TUNEL solution was configured with a sample of 5 μl TdT enzyme and 45 μl fluorescent labeling solution in a 12-well plate. Cortical neurons and SY5Y cells were inoculated on 12-well plates (50,000 cells/pore). After the experimental treatment, Cortical neurons and SY5Y cells were processed as follows: The cells were washed once with PBS, fixed with 4% paraformaldehyde for 30 minutes, washed once with PBS, permeabilized with 0.3% Triton x-100 at room temperature for 5 minutes, and washed once with PBS. After washing, the cells were incubated with TUNEL solution at 37 °C for 60 minutes and again washed once with PBS. Then, the cells were incubated with DAPI for 3 minutes, incubated with PBS once, and finally observed under a microscope. Apoptosis rate = number of dead cells/number of all cells.

### Western blot

The cortical neurons and SY5Y cells were inoculated in the plate. After the experimental treatment, tandem processing. To prepare the samples, the supernatant was discarded after treatment, the lysate was added at 100 ul/plate, and a protease inhibitor and phosphorylation inhibitor were added (1%). Then, the cells were cleaved on ice for 0.5 h, and the cells were scraped by using a cell brush and centrifuged for 15 min at 12000 r/min at 4 °C. After centrifugation, the supernatant was taken, and then we added 25 μl loading buffer per tube and boiled for 10 minutes. For the western blot, the following parameters were used: 10 μg per well protein loading, 80 V for electrophoresis, 200 mA transfer to a PVDF membrane. After completion of the transfer, the membranes were blocked with 5% milk, incubated with primary μ-calpain antibody (1:1000), CDK5 antibody (1:1000) and GAPDH antibody (1:1000) overnight at 4 °C, and washed with TBST 6 times. Then, the secondary antibody incubation was performed for 1 h, followed by washing with TBST 6 times and development.

### Immunofluorescence

Cortical neurons and SY5Y cells were inoculated on 12-well plates (50,000 cells/pore). After the experimental treatment, tandem processing. Cortical neurons and SY5Y cells were washed with PBS once, fixed with 4% paraformaldehyde for 0.5 h, washed with PBS once, permeabilized with 0.3% Triton x-100 in 5% BSA for 1 h, and incubated with AT8 antibody (1:500) and Map2 antibody (1:1000) overnight at 4 °C. The next day, the cells were washed with PBS three times, incubated with fluorescent secondary antibody for 1 h, washed with PBS three times, incubated with DAPI for 3-5 minutes, and incubated with PBS once prior to observation under a microscope. The AT8 positive rate of SY5Y cells was the number of AT8 positive cells/total number of cells × 100%.

### LPA ELISA

The samples were derived from cell culture medium and cell lysate. After the treatment, the cell medium was firstly collected. The cells were washed with PBS, and digested in lysis buffer containing 0.25% trypsin for 1 minute. The cell lysates were centrifuged for 5 minutes (800 r/min), took the cell pellet and stored in PBS. The protein concentrations in the supernatant were measured using the BCA protein detection kit (Thermo #23225), and then lysates of each sample were adjusted to the same level. Finally, the LPA level of each sample was measured using an ELISA kit according to the manufacturer’s instructions. The ELISA kit was performed according to the manufacturer’s instructions.

### Statistical analysis

All quantitative data are expressed as the mean ± standard deviation. Mann-Whitney U test (comparison between the two groups) and Kruskal-Wallis test (comparison between three or more groups) were used for statistical analysis, and then Bonferroni Post Hoc Test was subsequently used for comparison. *P* < 0.05 was considered statistically significant.

## Results

### LPA induces apoptosis of cortical neurons

**Fig. 1:**
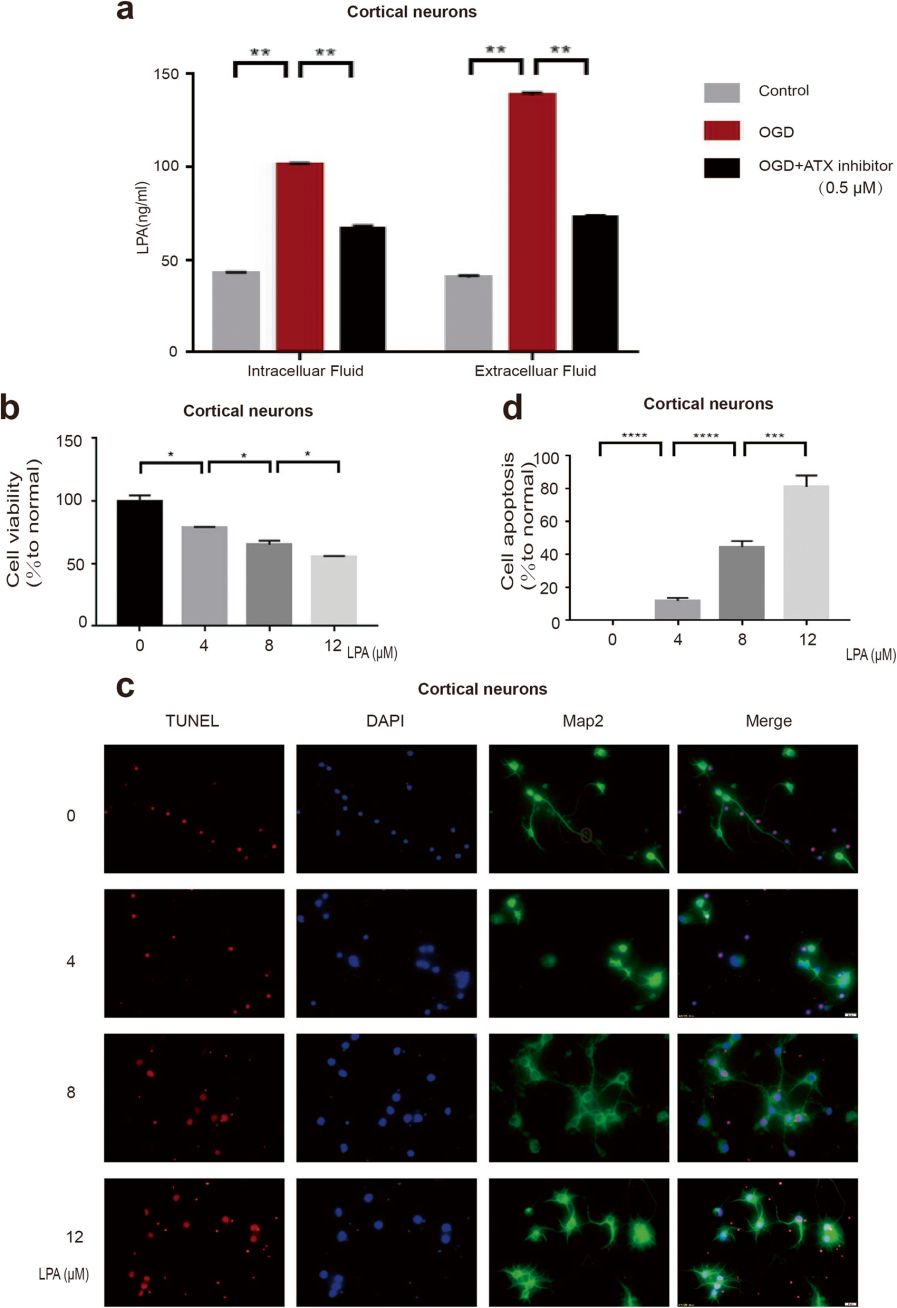
Changes in the LPA level during in vitro ischemia and neuronal cell death LPA induced. Detection of LPA levels in intracellular and extracellular fluid of cortical neurons by ELISA (**a**). CCK-8 was used to detect neuronal activity (**b**). The TUNEL assay was used to determine the apoptosis of cortical neurons at different LPA concentrations (**c**,**d**). The data are presented as the average ± S.E.M. from three or four independent cell experiments, *P < 0.05, **P < 0.01. ***P < 0.001, ****P < 0.0001

We first measured the LPA concentration during ischemia in primary cultured neurons. Using the in vitro OGD model, we found that LPA was increased in cortical neurons (Fig. 1a). To investigate the effect of LPA on cell death, CCK-8 and TUNEL were used to detect cell injury(1 μM LPA = 436.53 ng/ml LPA). CCK-8 analysis showed that LPA had a dose-dependent effect on cell viability, resulting in the loss of cortical neurons (Fig. 1b). TUNEL staining also showed that LPA induced apoptosis of neurons in a dose-dependent manner (Fig. 1c,d). We also confirmed this result in SY5Y cells (Supplementary data Fig. 1).

### Inhibition of LPA production alleviates neuronal injury induced by ischemia

**Fig. 2:**
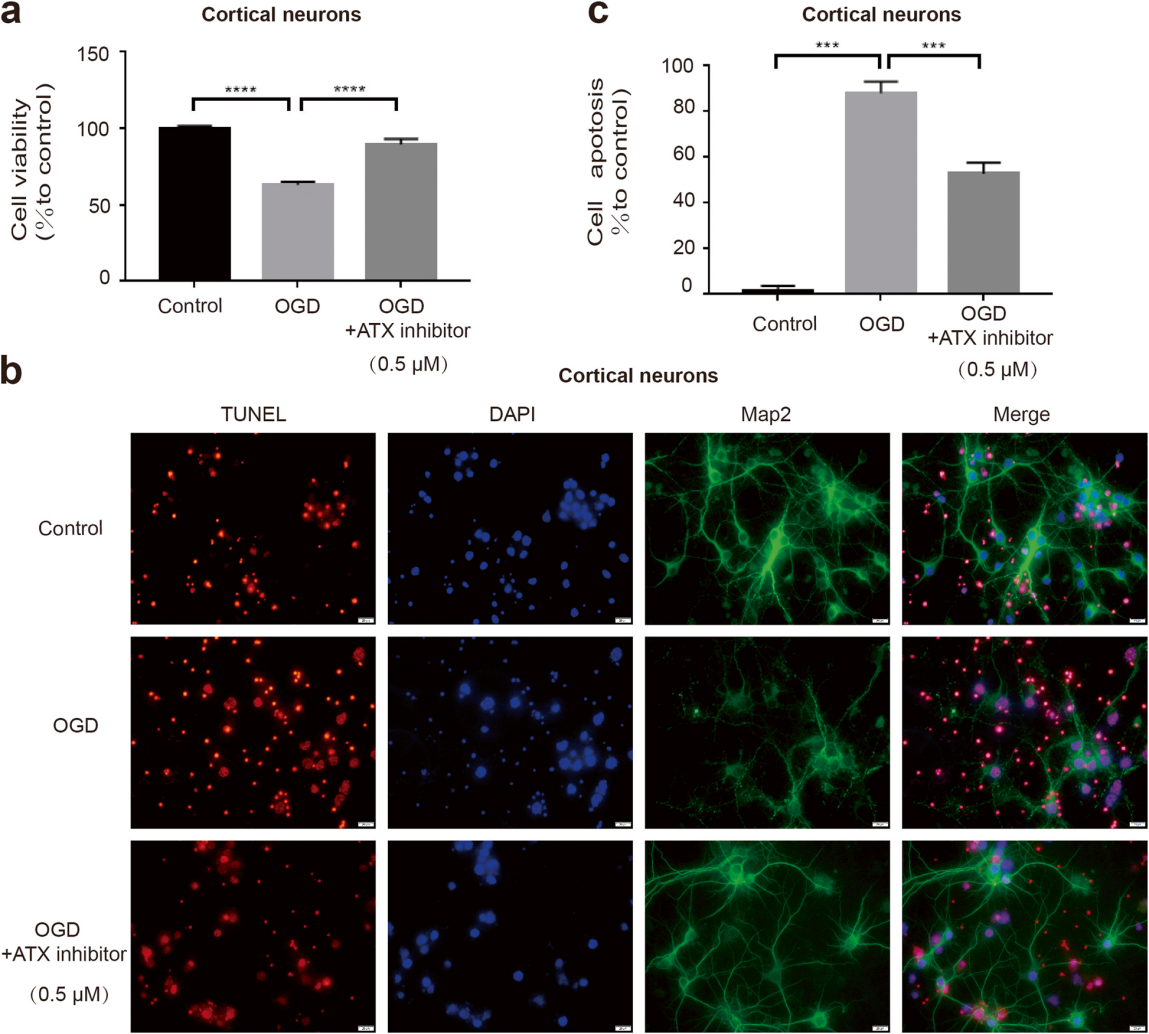
Blocking the production of LPA can reduce neuron death. CCK-8 detected the cell activity of neurons with or without OGD treatment as well as the cell activity of OGD-treated cortical neurons treated with an autotaxin inhibitor (**a**). TUNEL detected the apoptosis of cortical neurons with or without OGD treatment as well as apoptosis of OGD-treated cortical neurons treated with an autotaxin inhibitor (**b**,**c**). Data are presented as the mean ± S.E.M. from there independent cell experiments, ***P < 0.001,****P < 0.0001

To further investigate the role of LPA in neuronal death, an ATX inhibitor was used to prevent the elevation of LPA after ischemia (Fig. 1a-b). CCK-8 analysis of cortical neurons found that ATX inhibitor reduced OGD-induced injury (Fig. 2a). TUNEL staining also showed that ATX inhibitor significantly reversed cortical neuronal apoptosis induced by ischemia (Fig. 2b,c). These data suggest that LPA plays a role in ischemia-induced neuronal injury.

### LPA receptor inhibitors reduce neuronal damage

**Fig. 3:**
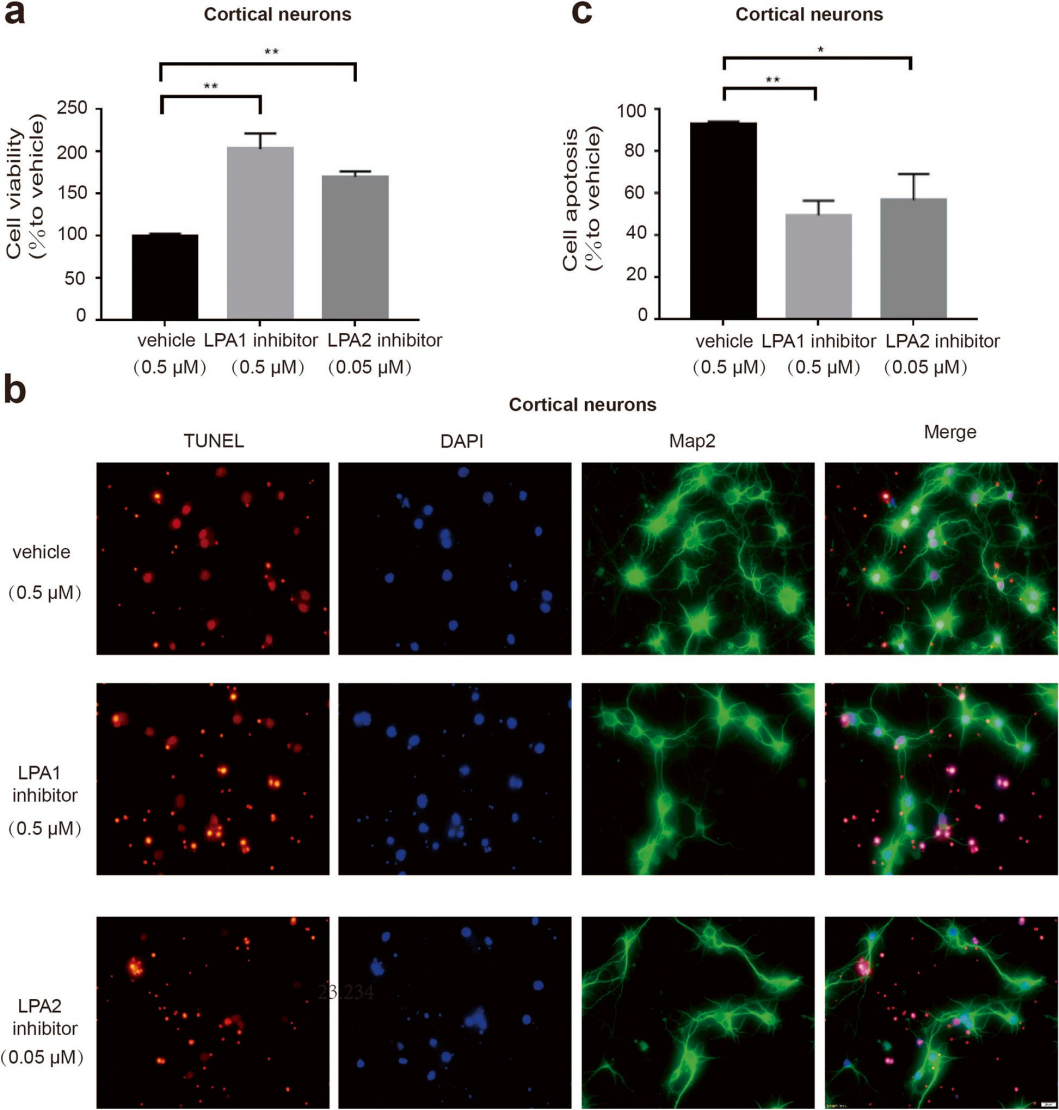
Blocking the LPA receptors reduces neuron death. CCK-8 detected the cell activity of neurons pretreated with LPA1 and LPA2 receptor inhibitors (**a**). TUNEL detected the apoptosis of neurons with the same treatment (**b**,**c**). Data are presented as the mean ± S.E.M. from there independent cell experiments, *P < 0.05. **P < 0.01

To further study the role of LPA in neuronal death, we pretreated the cells with LPA1 and LPA2 receptor inhibitors, respectively. CCK-8 analysis and the TUNEL assay showed that the damage of cortical neurons induced by LPA can be significantly improved by LPA1 and LPA2 receptor inhibition (Fig. 3a-c). We also confirmed this result in SY5Y cells (Supplementary data Fig. 2). These data suggest that LPA induces neuronal injury by activating its receptors.

### LPA promotes abnormal activity of CDK5 and phosphorylation of tau

**Fig. 4:**
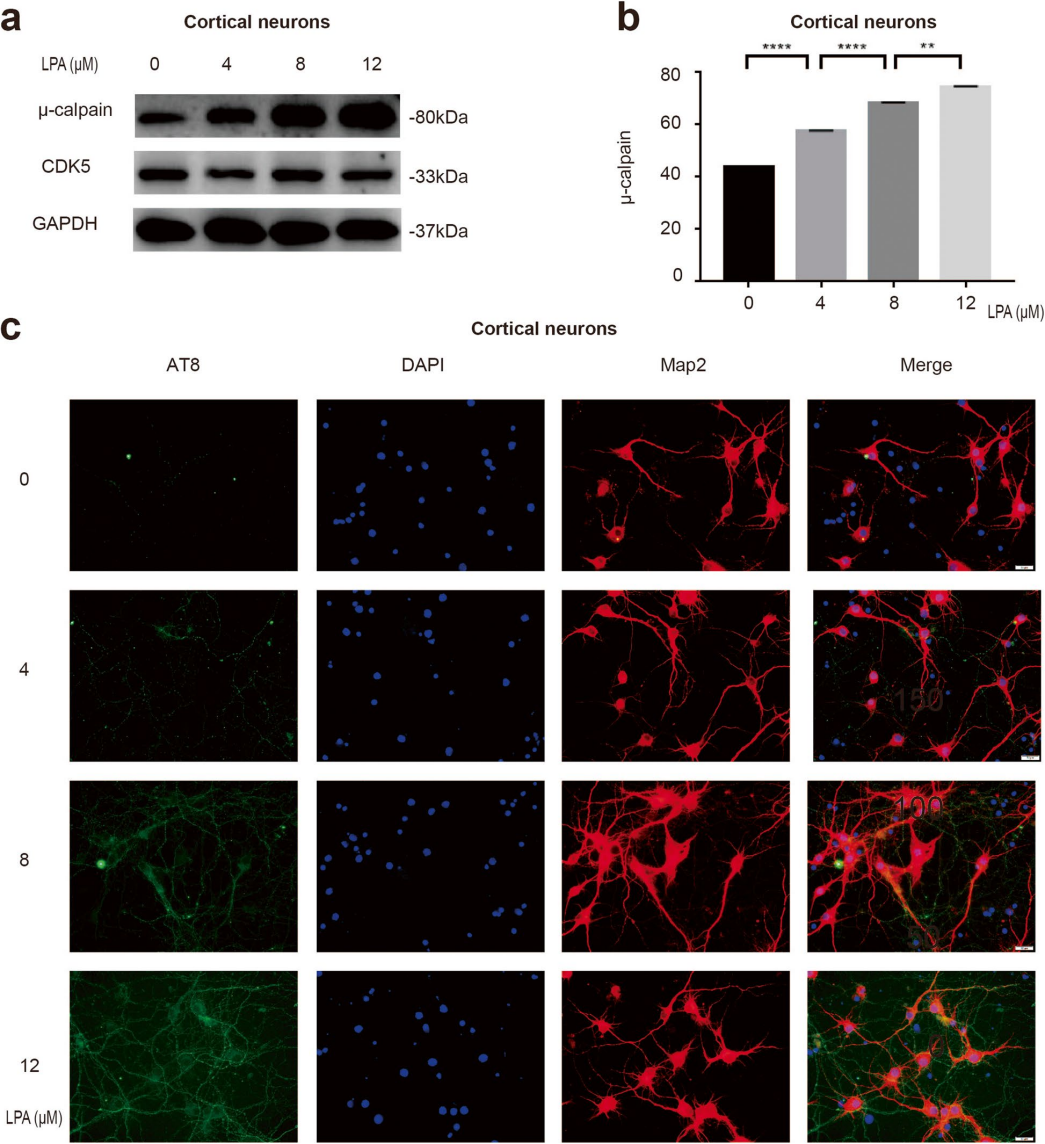
LPA can promote the expression of μ-calpain and AT8. Western blot detection of μ-calpain in cortical neurons treated with LPA (**a**,**b**). AT8 expression in cortical neurons after LPA treatment (**c**). Data are presented as the mean ± standard deviation from four independent cell experiments, **P < 0.01, ****P < 0.0001

To further investigate the role and mechanism of LPA-induced neuronal apoptosis and to test whether it could play a role in activating CDK5 activity, cortical neurons were treated with different concentrations of LPA. We found that LPA increased the levels of both μ-calpain and AT8 in cortical neurons in a dose-dependent manner (Fig. 4a-c). We also confirmed the effect of LPA on μ-calpain expression and tau phosphorylation in SY5Y cells (Supplementary data Fig. 3). Phosphorylation of tau by abnormally active CDK5 has been reported. μ-calpain can cut p35 to p25 and abnormally activate CDK5. These results indicate that LPA mediates the abnormal activation of CDK5 and the phosphorylation of tau protein.

### Inhibition of LPA production reverses the abnormal activation of CDK5 and the phosphorylation of tau

**Fig. 5:**
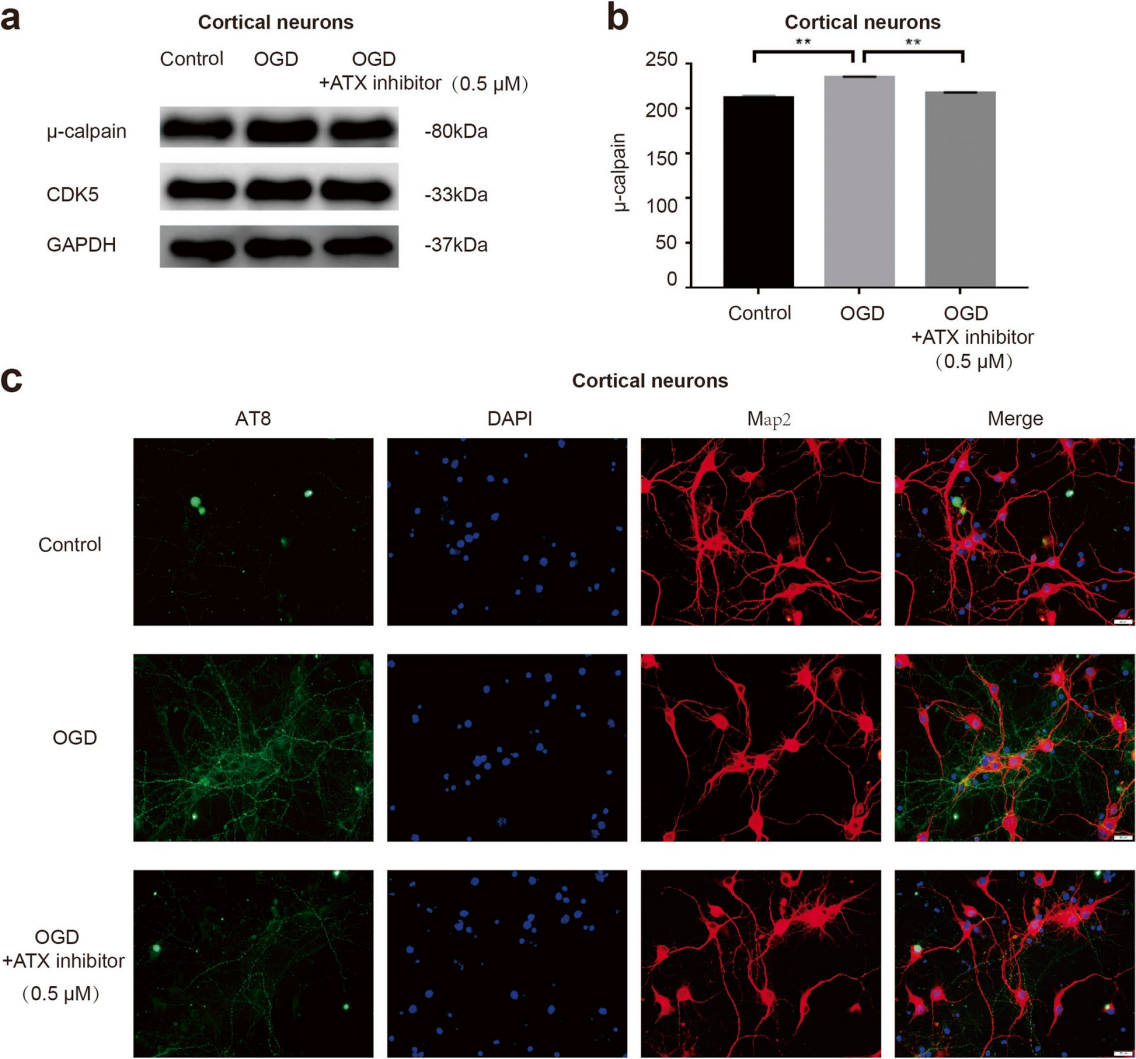
Blocking the production of LPA can reduce the expression of μ-calpain and AT8. Western blot measured μ-calpain of neurons treated with OGD with or without the autotaxin inhibitor (**a**,**b**). Immunofluorescence analysis of AT8 in neurons treated with OGD with or without the autotaxin inhibitor (**c**). Data are presented as the mean ± S.E.M. from three independent cell experiments, **P < 0.01

To further investigate the effect of LPA on CDK5 and AT8 after ischemia, we used an ATX inhibitor to block the production of LPA and detected the expression of μ-calpain and AT8. μ-calpain and AT8 were increased in cortical neurons in the OGD group compared with the control group, and the expression of μ-calpain and AT8 was decreased in the ATX inhibitor group compared with the OGD group (Fig. 5a-c), suggesting that LPA aggravates brain damage by activating CDK5 and thus phosphorylating tau.

### The effect of LPA receptor inhibitors on CDK5

**Fig. 6:**
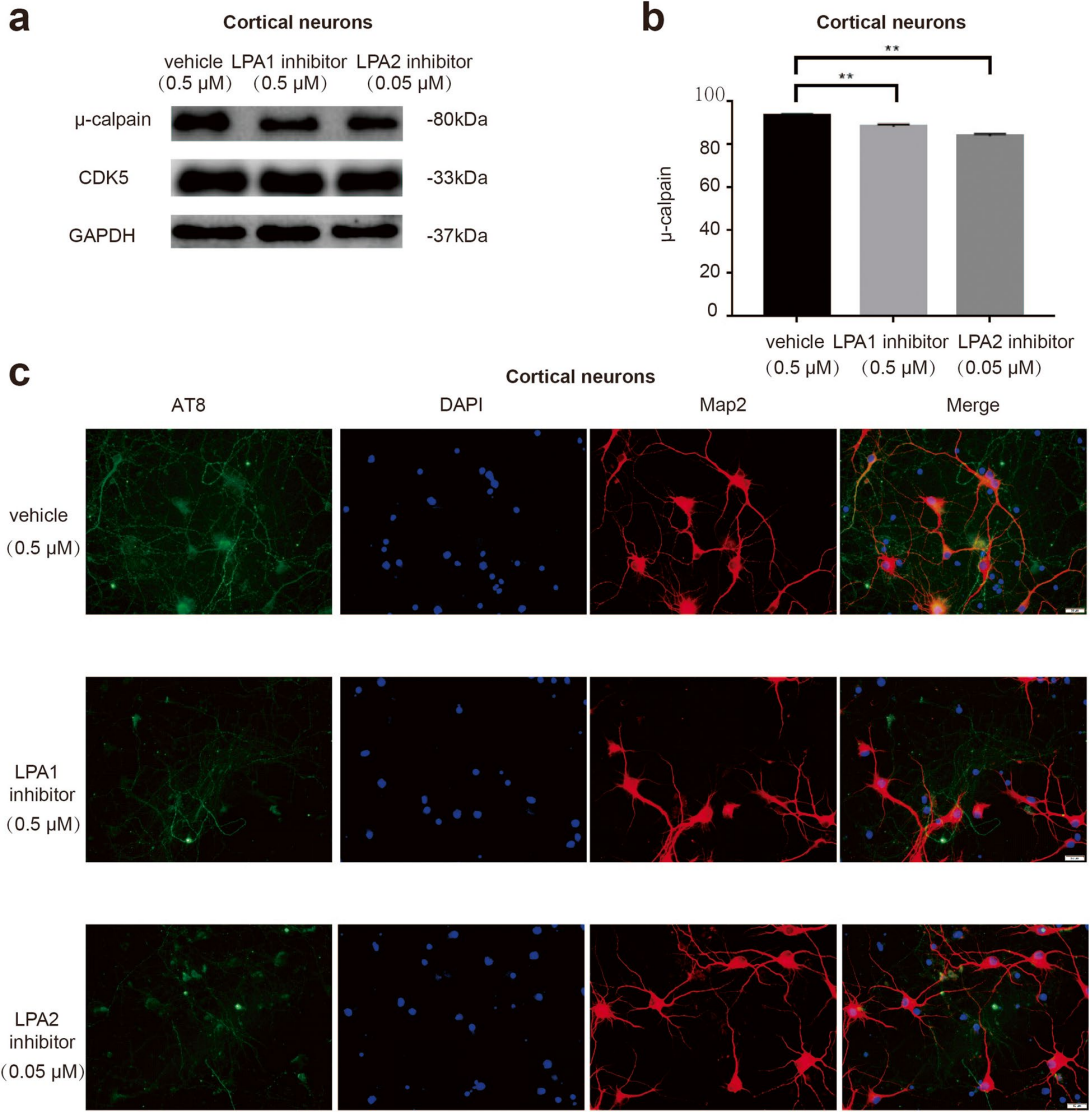
Blocking the LPA receptors can reduce the expression of μ-calpain and AT8. Western blotting measured μ-calpain in neurons pretreated with LPA1 and LPA2 receptor inhibitors (**a**,**b**). Immunofluorescence analysis of AT8 in neurons with the same treatment (**c**). Data are presented as the mean ± S.E.M. from three independent cell experiments, **P < 0.01

To further study the role and mechanism of LPA-induced neuronal apoptosis, we used LPA1 and LPA2 receptor inhibitors pretreatment in cortical neural and found that the LPA1 and LPA2 receptor inhibitors can reduce μ-calpain and AT8 expression in cortical neurons treated with LPA (Fig. 6a-c). We also confirmed the effect of LPA receptors on μ-calpain expression and tau phosphorylation in SY5Y cells (Supplementary data Fig. 4). The results show that LPA activates CDK5 through its two receptors, leading to tau phosphorylation, and thus mediating brain damage.

### Inhibition of CDK5 attenuates LPA-mediated tau phosphorylation and neuronal apoptosis

**Fig. 7:**
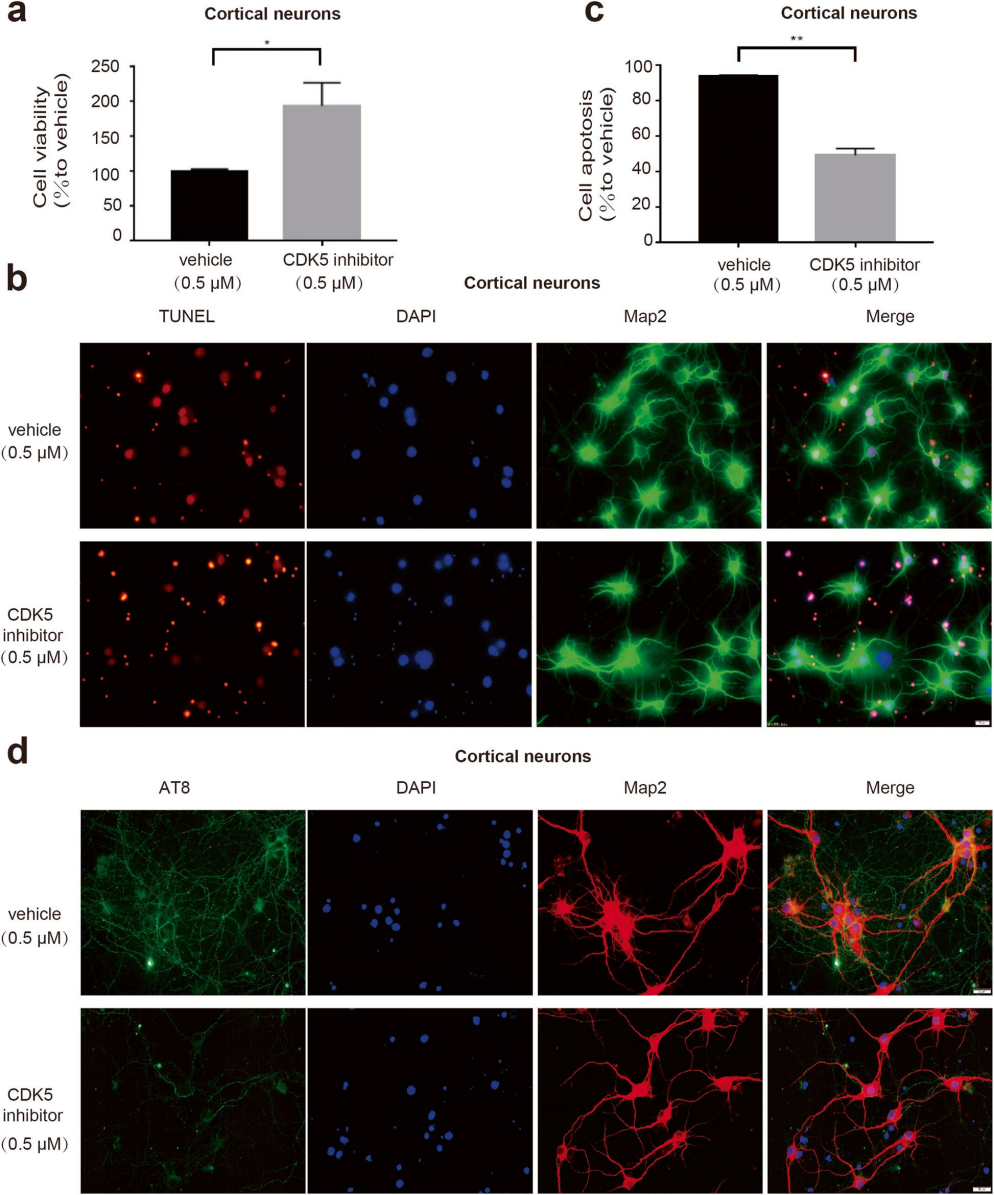
Blocking CDK5 can reduce the expression AT8 and reduce neuron death mediated by LPA. CCK-8 and TUNEL assays were used to determine the apoptosis of cortical neurons with or without CDK5 inhibitor (**a**-**c**). Immunofluorescence analysis of AT8 in neurons with or without CDK5 inhibitor (**d**). Data are presented as the mean ± S.E.M. from two independent cell experiments, *P < 0.05, **P < 0.01

To further study the role of LPA-induced neuronal apoptosis and its mechanism, we pretreated cortical neurons with CDK5 inhibitor. We found that CDK5 inhibitors reduced LPA-mediated apoptosis of cortical neurons (Fig. 7a-c) and reduce the LPA-mediated increase in AT8 expression (Fig. 7d). We also confirmed the effect of CDK5 inhibitor on cell apoptosis and tau phosphorylation in SY5Y cells (Supplementary data Fig. 5). The results showed that LPA leads to tau phosphorylation by activating CDK5, which mediates brain damage.

## Discussion

It has been reported that the LPA level is significantly increased in ischemic stroke, congenital hydrocephalus and multiple sclerosis [9, 10, 26], and the level of LPA receptor in the blood of patients with ischemic stroke is also increased [11]. Our study shows that LPA aggravates ischemia-reperfusion injury through an ATX-mediated pathway. LPA is increased in the brain and cerebrospinal fluid after injury [7, 27]. LPA is mainly derived from activated platelets [28]. However, other cell types such as endothelial cells, neurons, and glial cells are also involved in the production of LPA [29]. Previous studies have demonstrated that LPA mediates a variety of cell functions, including cell proliferation, cell migration, cell survival and cytoskeletal recombination [30, 31]. In LPA receptors, LPA1 mediates cell survival, proliferation, adhesion, migration, immune function and myelin formation. LPA2 and LPA1-mediated effects are similar and complementary [31–33]. LPA1 receptor mainly exists in neurons and glial cells in the brain, while LPA2 receptor is highly expressed in neurons [3]. We hypothesized that LPA activates the downstream pathway through its LPA1 and LPA2 receptors to induce neuronal apoptosis after ischemia in vitro, but the signaling pathway of this process is still unclear.

CDK5 is a proline-oriented Ser/Thr protein kinase which can phosphorylate tau [34, 35], and μ-calpain can truncate p35 into p25, resulting in abnormal activation of CDK5 and the death of neurons associated with a variety of neurodegenerative diseases [16]. Calpain is a ubiquitous Ca^2+^-dependent cysteine protease, the activation of μ-calpain need 3-50 μM calcium [36]. Our study shows that LPA promotes aberrant activation of CDK5 by increasing μ-calpain. Previous reports found that LPA activates calpain by binding to the LPA1 receptor [37, 38]. The possible mechanism is that LPA activates the Ca^2+^ signal transduction process, leading to an increase in Ca^2+^ content and Ca^2+^ overload, and thus calpain activation [39, 40]. Previous studies have shown the time-dependent increase in p25 levels under hypoxia, which is consistent with neuronal death. Compared with neurons expressing GFP, the death of neurons expressing p25 is increased. At the same time, importantly, inhibition of CDK5 kinase activity can reduce hypoxia-mediated neuronal death. Calpain inhibitors can reduce tau phosphorylation [41, 42], which is consistent with our study.

It also has been reported that during ischemia-reperfusion injury, tau phosphorylation increases in the ischemic cortical region [43]. Tau phosphorylation is considered to be a key pathological process mediating synaptic injury and neuronal loss [44, 45]. Our study shows that abnormal CDK5 activation increases the expression of downstream phosphorylated tau protein, which induces neuronal cell death. This is consistent with previous studies on the changes of p25/CDK5 and tau phosphorylation in ischemia-reperfusion injury [46, 47].

We found that after ischemic injury, LPA increased, CDK5 activation was abnormal, and tau phosphorylation increased. Blocking the production of LPA and inhibiting LPA1 and LPA2 receptors reduced the abnormal activation of CDK5 and the production of phosphorylated tau in neurons. AM095 used in this experiment is a specific pharmacological inhibitor of LPA1 [48, 49]. LPA2 receptor antagonist (HY-18075) is a selective inhibitor of LPA2 that was evaluated in vitro for its inhibition of LPA2-mediated Erk activation [50]. According to the literature, the combination of antagonists may enhance the inhibitory effect. In experiments in which LPA1 and LPA2 receptor inhibitors reduce μ-calpain expression in LPA-treated neurons, the statistical results of μ-calpain showed that the change was less than 10%. But the experimental phenomenon of inhibiting the phosphorylation of tau protein was obvious, and the change of μ-calpain in SY5Y cells exceeded 10%, these experimental phenomena have some corroborative significance. These results showed that LPA promoted abnormal activation of CDK5 in the pathological process of ischemic stroke. Combined with the neuronal apoptosis experiment, LPA promoted neuronal apoptosis in a concentration-dependent manner, ATX inhibitors and LPA1 and LPA2 receptor inhibitors could reduce neuronal apoptosis.

We also found that CDK5 inhibitors could reduce phosphorylated tau protein and neuronal apoptosis mediated by LPA. Roscovitine is a non-specific CDK inhibitor that inhibits CDK5, Cdc2 and CDK2. However, the use of roscovitine also reduced the phosphorylation level of tau protein and neuronal apoptosis, we guessed that it was mainly mediated by the abnormal activation pathway of CDK5 caused by μ-calpain. At the same time, it is also possible to mediate neuronal apoptosis through other pathways. In the follow-up study, the expression level of μ-calpain and the levels of other CDK activation-related substances should be explored to further explore the specific mechanism. Although some of the experimental approaches in this manuscript have limitations, these observations suggest the idea that increased LPA after ischemic stroke promotes abnormal activation of CDK5 and tau phosphorylation, which in turn induces neuronal cell death.

Interestingly, in the experiment of neuronal apoptosis, we found that some other brain cells, including glial cells, also have apoptosis. Previous studies have shown that LPA mediates glial inflammation [51]. The potent survival effect of LPA on Schwann cells is mediated through the pertussis toxin (PTX)-sensitive G(i/o)/phosphoinositide 3-kinase (PI3K)/Akt signaling pathway, and LPA also induces Apoptosis in certain cells, such as myeloid progenitor cells, hippocampal neurons and PC12 cells, involves activation of Rho-dependent pathways and caspase cascades [52]. It has also been shown that neuronal p25 rapidly and strongly triggers neurodegeneration and microgliosis in the hippocampus and cortex, but neurodegeneration does not involve the phosphorylation of the protein tau or the production of amyloid peptides [53]. But the evidence still fails to indicate whether apoptosis of other brain cells is related to the LPA-CDK5-Tau pathway.

In the present study, we conjectured that CDK5-Tau pathway mediates the activity of LPA in vitro. It has been reported that the CDK5 activation leads to neuronal apoptosis in an in vivo ischemia-reperfusion injury model. The total infarcted area was significantly reduced in CDK5 conditional knock-out mice, which is consistent with our results. Interestingly, the neuroprotective effect of CDK5 conditional knock-out is evident in the striatum and does not extend to the cortex in MCAO model [17]. The likely reason is that the striatum produces more p25 and the cortex has more collateral blood vessels than the striatum. However, the role of CDK5-Tau pathway in LPA-mediated neuronal injury needs to be further verified in animal experiments. However, this study is the initial evidence for the molecular mechanisms of ischemic injury, which may lay the foundation for the development of more effective clinical treatments.

## Supplementary Information

**Additional file 1: Figure 1.** Changes in the LPA level during in vitro ischemia and LPA induces cell death. Detection of LPA levels in intracellular and extracellular fluid of SY5Y cells by ELISA (a). CCK-8 was used to detect cell activity of SY5Y cells (b). The TUNEL assay was used to determine the apoptosis of SY5Y cells at different LPA concentrations (c,d). The data are presented as the average ± S.E.M. from three or four independent cell experiments, **P < 0.01.***P < 0.001, ****P < 0.0001.

**Additional file 2: Figure 2.** Blocking the LPA receptors reduces cell death. CCK-8 detected the cell activity of SY5Y cells pretreated with LPA1 and LPA2 receptor inhibitors (a). TUNEL detects the apoptosis of SY5Y cells with the same treatment (b,c). Data are presented as the mean ± S.E.M. from there independent cell experiments, *P < 0.05. **P < 0.01. ****P < 0.0001.

**Additional file 3: Figure 3.** LPA can promote the expression of μ-calpain and AT8.Western blot analysis of μ-calpain in SY5Y cells after LPA treatment (a,b). AT8 expression in SY5Y cells treated with LPA (c,d). Data are presented as the mean ± standard deviation from four independent cell experiments, *P < 0.05, **P < 0.01,***P < 0.001.

**Additional file 4: Figure 4.** Blocking the LPA receptors can reduce the expression of μ-calpain and AT8. Western blotting measured μ-calpain in SY5Y cells pretreated with LPA1 and LPA2 receptor inhibitors (a,b). Immunofluorescence analysis of AT8 in SY5Y cells with the same treatment (c). Data are presented as the mean ± S.E.M. from three independent cell experiments, *P < 0.05, **P < 0.01, ****P < 0.0001.

**Additional file 5: Figure 5.** Blocking CDK5 can reduce the expression AT8 and neuron death mediated by LPA. CCK-8 and TUNEL assays were used to determine the apoptosis of SY5Y cells with or without CDK5 inhibitor (a-c). Immunofluorescence analysis of AT8 in SY5Y cells with or without CDK5 inhibitor (d,e). Data are presented as the mean ± S.E.M. from independent cell experiments, *P < 0.05, **P < 0.01.

## Data Availability

The datasets used and/or analysed during the current study available from the corresponding author on reasonable request.
