# Supplementary material for: The LPA-CDK5-tau pathway mediates neuronal injury in an in vitro model of ischemia-reperfusion insult
Source: BMC Neurol. 2022 May 2;22:166. doi: 10.1186/s12883-022-02694-2 (PMC9059403; doi:10.1186/s12883-022-02694-2)
Supplement: Supplementary file 1 — Additional file 1: Figure 1. Changes in the LPA level during in vitro ischemia and LPA induces cell death. Detection of LPA levels in intracellular and extracellular fluid of SY5Y cells by ELISA (a). CCK-8 was used to detect cell activity of SY5Y cells (b). The TUNEL assay was used to determine the apoptosis of SY5Y cells at different LPA concentrations (c,d). The data are presented as the average ± S.E.M. from three or four independent cell experiments, **P < 0.01.***P < 0.001, ****P < 0.0001. [file 12883_2022_2694_MOESM1_ESM.docx]

**Supplementary data**


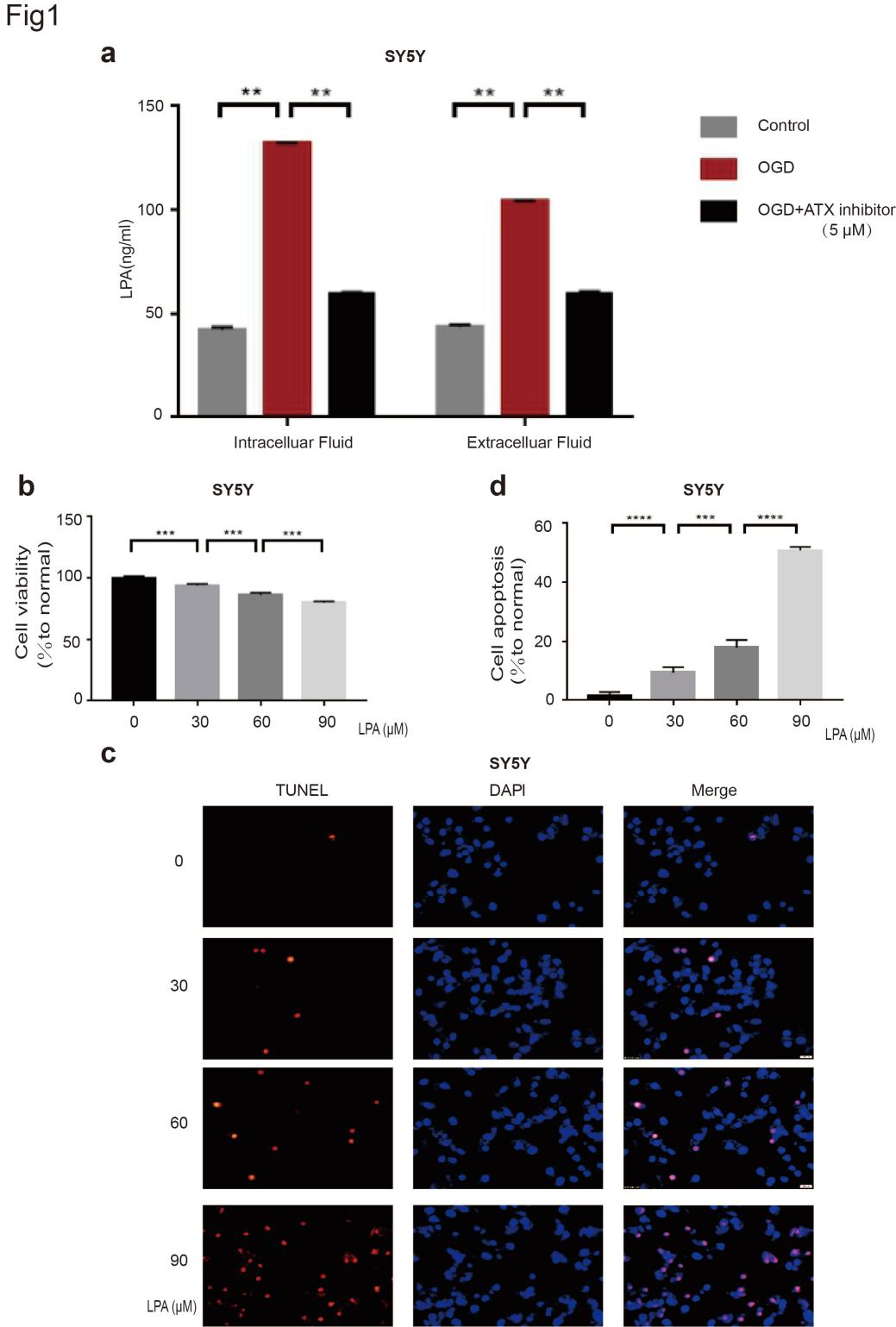


Fig. 1. Changes in the LPA level during in vitro ischemia and LPA induces cell death. Detection of LPA levels in intracellular and extracellular fluid of SY5Y cells by ELISA (a). CCK-8 was used to detect cell activity of SY5Y cells (b). The TUNEL assay was used to determine the apoptosis of SY5Y cells at different LPA concentrations (c,d). The data are presented as the average ± S.E.M. from three or four independent cell experiments, **P < 0.01.***P<0.001, ****P<0.0001.


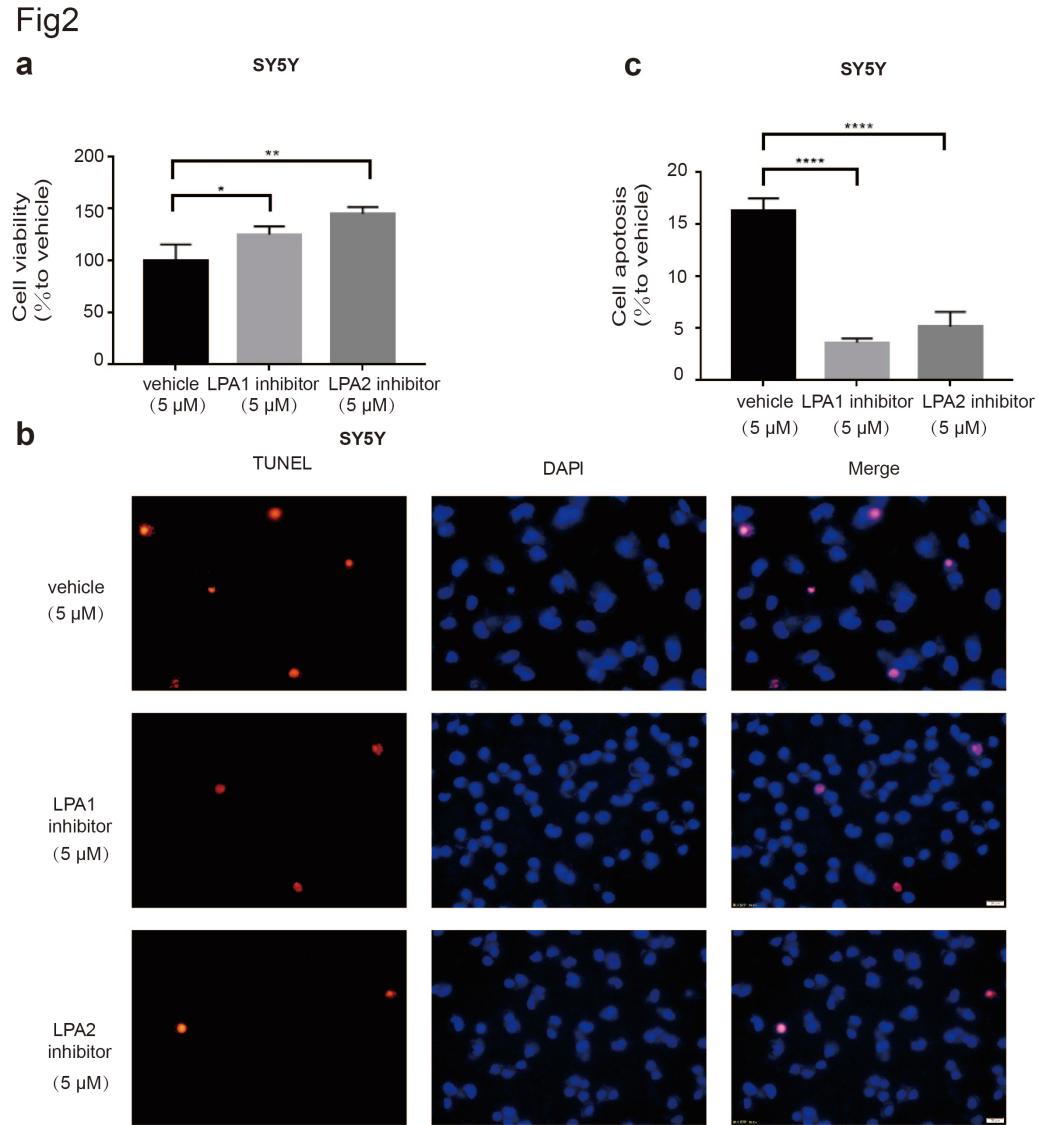


Fig. 2. Blocking the LPA receptors reduces cell death. CCK-8 detected the cell activity of SY5Y cells pretreated with LPA1 and LPA2 receptor inhibitors (a). TUNEL detects the apoptosis of SY5Y cells with the same treatment (b,c). Data are presented as the mean ± S.E.M. from there independent cell experiments, *P < 0.05. **P < 0.01. ****P < 0.0001.


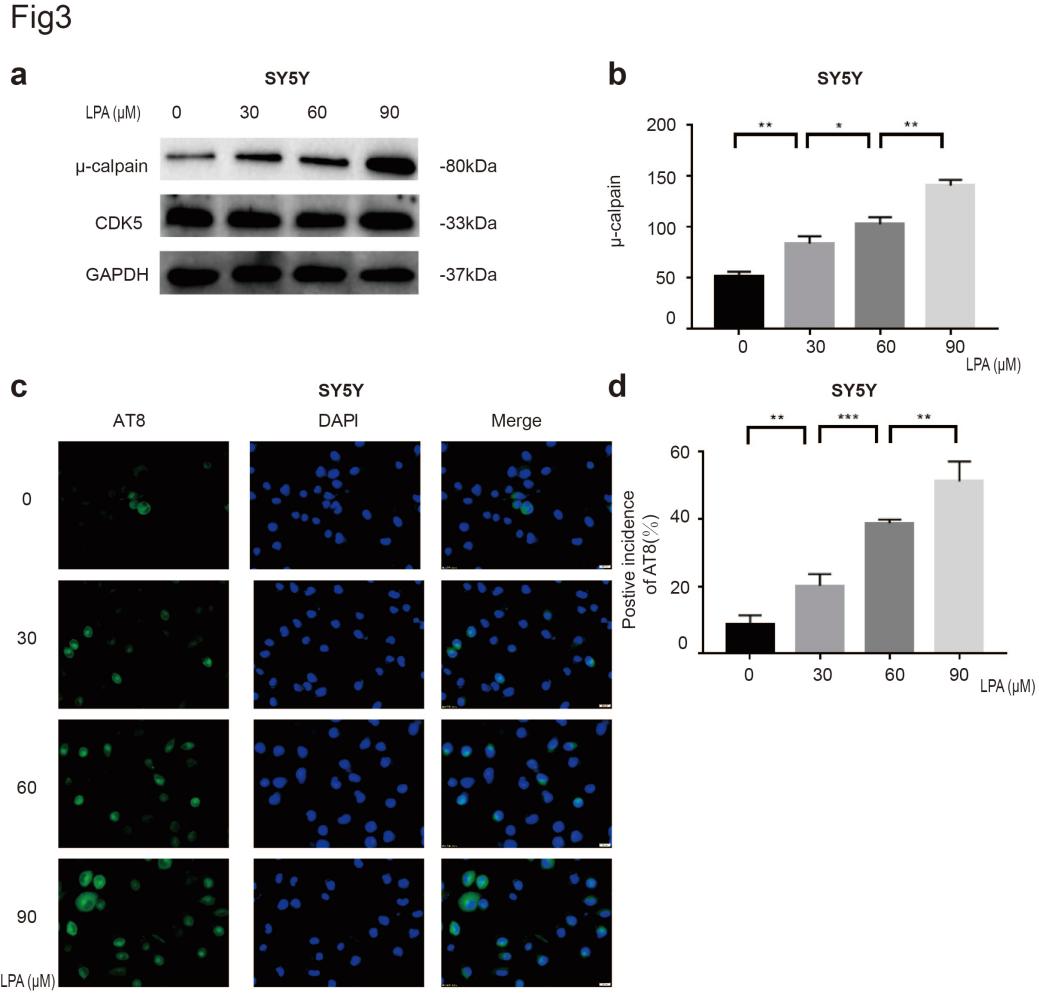


Fig. 3. LPA can promote the expression of μ-calpain and AT8.Western blot analysis of μ-calpain in SY5Y cells after LPA treatment (a,b). AT8 expression in SY5Y cells treated with LPA (c,d). Data are presented as the mean ±standard deviation from four independent cell experiments, *P < 0.05, **P < 0.01,***P<0.001.


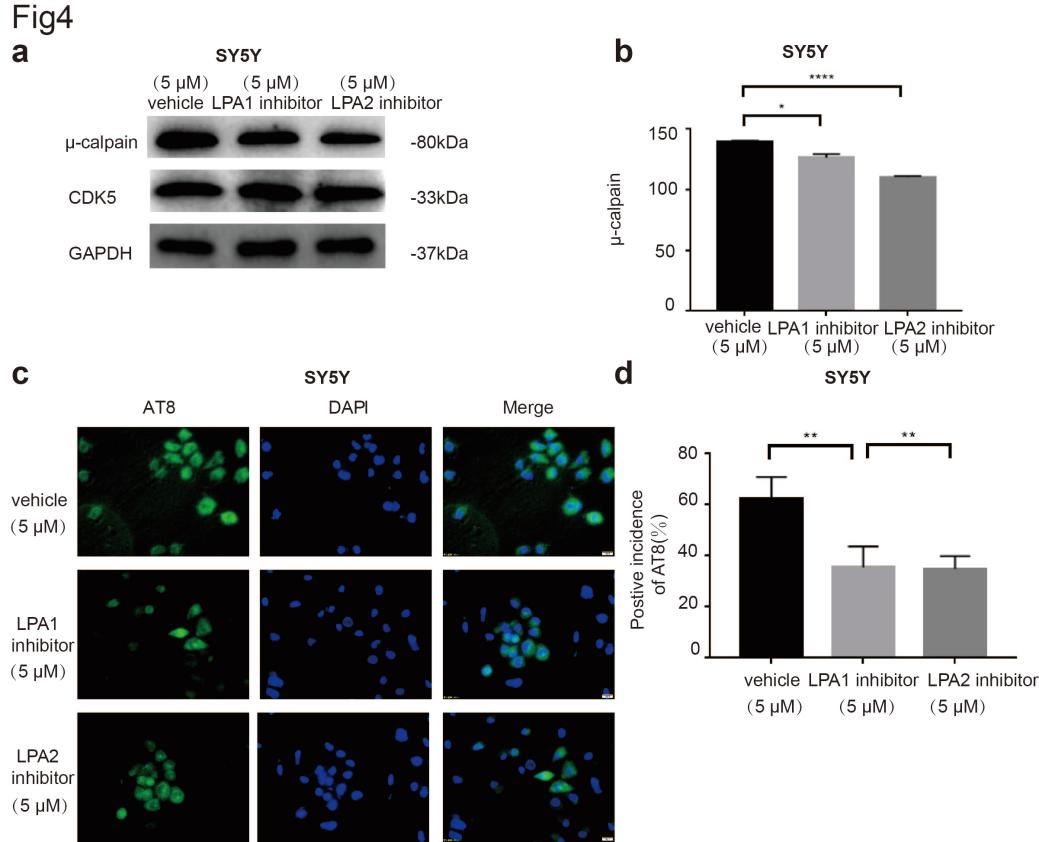


Fig. 4. Blocking the LPA receptors can reduce the expression of μ-calpain and AT8. Western blotting measured μ-calpain in SY5Y cells pretreated with LPA1 and LPA2 receptor inhibitors (a,b). Immunofluorescence analysis of AT8 in SY5Y cells with the same treatment (c). Data are presented as the mean ± S.E.M. from three independent cell experiments, *P < 0.05, **P < 0.01, ****P<0.0001.


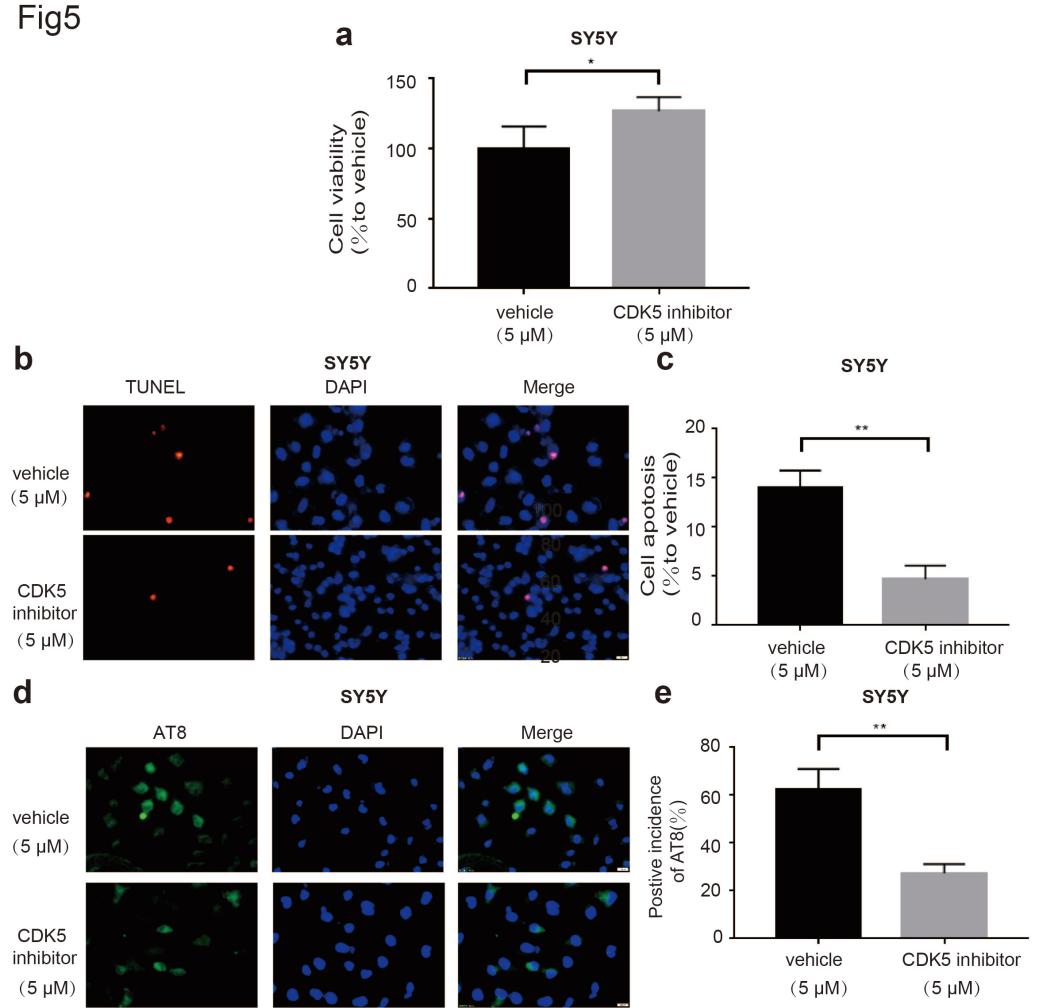


Fig. 5. Blocking CDK5 can reduce the expression AT8 and neuron death mediated by LPA. CCK-8 and TUNEL assays were used to determine the apoptosis of SY5Y cells with or without CDK5 inhibitor (a-c). Immunofluorescence analysis of AT8 in SY5Y cells with or without CDK5 inhibitor (d,e). Data are presented as the mean ± S.E.M. from independent cell experiments, *P < 0.05, **P < 0.01.
